# An ethical assessment model for digital disease detection technologies

**DOI:** 10.1186/s40504-017-0062-x

**Published:** 2017-09-20

**Authors:** Kerstin Denecke

**Affiliations:** Bern University of Applied Sciences, Bern, Switzerland

## Abstract

Digital epidemiology, also referred to as digital disease detection (DDD), successfully provided methods and strategies for using information technology to support infectious disease monitoring and surveillance or understand attitudes and concerns about infectious diseases. However, Internet-based research and social media usage in epidemiology and healthcare pose new technical, functional and formal challenges. The focus of this paper is on the ethical issues to be considered when integrating digital epidemiology with existing practices. Taking existing ethical guidelines and the results from the EU project M-Eco and SORMAS as starting point, we develop an ethical assessment model aiming at providing support in identifying relevant ethical concerns in future DDD projects. The assessment model has four dimensions: user, application area, data source and methodology. The model supports in becoming aware, identifying and describing the ethical dimensions of DDD technology or use case and in identifying the ethical issues on the technology use from different perspectives. It can be applied in an interdisciplinary meeting to collect different viewpoints on a DDD system even before the implementation starts and aims at triggering discussions and finding solutions for risks that might not be acceptable even in the development phase. From the answers, ethical issues concerning confidence, privacy, data and patient security or justice may be judged and weighted.

## Introduction

Digital epidemiology, also referred to as digital disease detection (DDD), successfully provided methods and strategies for using information technology to support infectious disease monitoring and surveillance or understand attitudes and concerns about infectious diseases. It starts from developments such as the widespread availability of Internet access or digital devices and online sharing platforms, which continuously produce large amounts of data. Often, those technologies are collecting data without a public health objective. For example, instant messaging, discussion groups or social networks are increasingly recognised as valuable sources of public health alerts since they are sources of first-hand information. It has been proven that awareness of diseases achieved through such observations can influence people’s behaviour and reduce the risk of an outbreak and the number of infected people [FUN09]. However, Internet-based research and social media usage in epidemiology and healthcare provide new technical, functional and formal challenges. Technical challenges include the increasing need for hardware and technologies that can process large amounts of data and technologies for efficiently identifying the relevant pieces in data sets. Functional challenges comprise the need for user- and use cases specific graphical user interfaces, for personalization methods that filter the gathered information in order not to overwhelm users with irrelevant information. Formal problems include quality and reliability of content, payment models as well as ethical and legal issues. The focus of this paper is on the ethical issues to be considered when integrating digital epidemiology with existing practices. Taking existing ethical guidelines and the results from the EU project M-Eco (Denecke et al. 2013) and the binational project SORMAS (Adeoye et al. 2017) as starting point, we develop an ethical assessment model aiming at providing support in identifying relevant ethical concerns in DDD projects. The model supports in becoming aware, identifying and describing the ethical dimensions of a technology or use case and in identifying the ethical issues on the technology use from different perspectives. It can be applied in an interdisciplinary meeting to collect different viewpoints on a DDD system even before the implementation starts and aims at triggering discussions and finding solutions for risks that might not be acceptable. From the answers, ethical issues concerning confidence, privacy, data and patient security or justice may be judged and weighted.

First, we will introduce the topic of ethics in public health and will summarize previous work on ethical issues in the context of digital health (section 2). The ethical assessment model that is introduced in section 4 is a result of the work in two projects. Therefore, the projects M-Eco and SORMAS are introduced in section 3. Lessons learnt from these projects with respect to ethical issues will be summarized. Finally, we will apply the model to the two projects for identifying ethical issues. The paper finishes with conclusions and future work.

## Ethics in public health and digital epidemiology

According to Oxford Dictionaries,^1^ ethics is defined as “moral principles that govern a person’s behavior or the conducting of an activity”. In other words, ethics is defined as discipline dealing with what is good and bad and with moral, duty and obligation. This broad definition becomes more specific when distinguishing *public health ethics* from *medical ethics*. *Medical ethics* concentrates on the relationship between patients and doctors. In contrast, *public health ethics* 2 deals with the specific moral questions of public actions for disease prevention, life elongation, or psychological and physical well-being. The specific and unique perspective of public health is its population perspective. Even though ethics is not a new topic in medicine, it is specific in public health. Holland claims that the specific challenge in public health ethics is the dilemma between protecting and promoting the health of populations and the risk of causing individual harm and costs (Holland 2014).

The oldest framework for addressing ethical issues in the context of medicine are provided by the Helsinki Declaration (Bourne 2015). According to these guidelines, it is necessary to get the patient consent before involving him or his data into a study. Patients that are unable to give consent need to be protected at any time. Each clinical research project need to be approved by an independent ethics committee. The well-being of the patient has a higher priority than the interest of science and research results from unethical experiments should not be published (Declaration of Helsinki 2013). It is clear that in the context of public health and in particular this holds true for DDD technologies, these guidelines cannot be directly applied: For example, we cannot always ask for patient consent in the context of DDD and health monitoring.

With the increasing availability of web technologies and health-related web content as well as the use of social media in the context of (public) health, researchers started to consider the critical perspectives of such digital health technologies including the ethical issues. The issue of how ethical principles can be applied to online health research has provided a challenge to researchers. Ethical and legal concerns regarding collection of data from social networks have been explored in a handful of articles and legal cases (Flicker et al. 2004; Moreno et al. 2008; Zimmer 2010). Bond et al. (2013) summarised the ethical issues that researchers should consider when researching with social media data in health contexts. Conducting research on social media sites requires deliberate attention to consent, confidentiality, and security. Beauchamp and Childress (2001) introduced three main principles for medical ethics that are autonomy, well-fare, and justice. When applying these principles in the context of digital disease detection, we need to consider that each person should have the right to decide about the usage of data and information concerning their private life – it is their right of informational self-determination. However, this can often not be realised in the public health context.

It can be seen that existing frameworks and guidelines for ethical concerns in healthcare can be applied to DDD technologies only to a limited extent. Anyway, they provide main aspects regarding ethics to be considered in the development of DDD technologies: Privacy, responsibility and expressiveness. As for other media use in healthcare, respecting privacy of individuals is important. However, there is responsibility that concerns weighting individual rights and benefits of DDD. Digital disease detection has a public function that is to improve health at population level. Ethical considerations concern:

- How can big data be utilized for the common good whilst respecting individual rights and liberties?
- What are the acceptable trade-offs between individual rights and the common good?
- How do we determine the thresholds for such trade-offs?

Expressiveness is a new issue and specific to digital diseases detection. With Internet data to be used for disease surveillance, we gain a source of unique information. However, its reliability needs to be carefully assessed; then, data could extend common clinical data. In previous work, we found out through a questionnaire that experts agree that health organisations should react when some hint to a public health problem is detected through social media monitoring (Denecke 2014). But they should not only rely upon that data. The interpretive value of social media data depends on the data analysis process. The data needs to be verified and corroborated with confirmed medical data to judge the interpretative value. Context-sensitive understanding of ethical obligations may reveal that some data uses that may not be acceptable within corporate activity (e.g. user profiling, data sharing with third parties) may be permissible for public health purposes.

## Projects in digital epidemiology

In the last years, multiple projects have been established that exploit internet data for DDD purposes (e.g. HealthMap (Brownstein et al. 2008; MediSys Linge et al. 2010)) or that exploit new upcoming cloud technology and mobile devices for supporting disease outbreak management. Even crisis communication is realized through social networks and social media (Holmes 2016). In this section, we will introduce two of those completed projects, M-Eco and SORMAS as examples for systems for DDD technologies. They have been chosen as starting point for the development of the ethical assessment model in this paper, since the author substantially contributed to these projects and thus, is aware for the technologies. From the data sources and underlying technologies, M-Eco is comparable with projects such as HealthMap that are still publishing monitoring results online.

### The medical ecosystem (M-eco)

The EU-funded project M-Eco: Medical Ecosystem was conducted between 2010 and 2012 with seven project partners from Austria, Italy, Germany, Czech Republic and Denmark, including the German health organisation Robert Koch Institute and with support of representatives of various health organisations including the World Health Organisation, European Center of Disease Prevention and control and Institute de Veille Sanitaire. In this section, we briefly summarize the architecture of the M-Eco system, its functionalities and report on experiences in evaluating and testing. The M-Eco system could so far not been established into regular use by health organisations. More details about the technology and studies can be found in papers by Denecke et al. (2013) and Velasco et al. (2014]).

The M-Eco system was intended to support in health monitoring during mass gathering events in a cross-country setting and in health monitoring on a national level. It monitored social media, TV, radio and online news and aggregated relevant content of these sources into signals. Signals pointed the user to relevant information and their sources which allowed to analyse its relevance and need for interaction through health officials. Automatically generated time series supported in monitoring disease activity over a longer time period. Tag clouds summarized the related information in a visual manner and supported navigation through signals. The plotting of signals to geographic maps allowed to localize disease outbreaks.

To realize these functionalities, the M-Eco system consists of a set of web services that cover 1) content collection, 2) signal generation, 3) user modelling and recommendation as well as 4) visualization in a user interface. The services work in a pipeline fashion and are triggered automatically four times a day.

The information database of the system is filled continuously by collecting data from various sources by means of web crawling and streaming APIs (e.g. the Twitter API). The collection focuses on broadcast news from TV and radio, news data from MedISys (Linge et al. 2010), and social media content from blogs, forums and Twitter. The TV and radio data is collected via satellite and transcribed to written text by SAILs Media Mining Indexing System (Backfried et al. 2012). About 1300 names of symptoms and diseases were used as keywords for collecting data extended by existing language resources such as WordNet, GermaNet, or the OpenOffice thesaurus. The data is tokenized and part-of-speech-tagged by the Tree Tagger and parsed by the Stanford Parser. All texts are also semantically annotated with geo-tags, disease or symptom tags and temporal expressions as well as with information on the affected organism.

The event detection and signal generation component exploits the annotated texts to generate signals. A signal is a hint to some anomalous event. Signals are produced with associated information on the disease or symptom the signal is referring to and a location that has been extracted for that signal. For all relevant sentences, i.e. sentences that match predefined keywords or expressions, entity pairs (location, disease) are exploited to produce time series for each entity pair occurring in sentences of texts published within one week. The time series provide the input for statistical methods for signal generation, CUSUM and Farrington. These two statistical methods have originally been developed for indicator-based surveillance (Hoehle 2007). The recommendation component gets as input the generated signals and either selects those that are of interest for a user according to his profile or ranks the signals appropriately. The component also supports users with personalized presentation options (e.g., tag clouds, list of recommendations) that are visualized in the user interface.

The M-Eco system results were analysed in several studies (Denecke et al. 2013; Velasco et al. 2014). They revealed characteristics of social media that are relevant for disease surveillance. First, the texts that contributed to signals rated as relevant by the epidemiologist often linked to media reports or so-called secondary reports. This experience let conclude that there might be a trend in social media whereby users tend to write less often about their personal specific symptoms, but most often forward information from reliable sources such as news sites, or prevention efforts from authorities. Second, most signals were generated from Twitter data. The volume of relevant Twitter data that is processed by the system is much higher than from any other source considered as input. Contrasting the initial expectation, the signals were not generated from clustered reports on personally reported symptoms, but on news reports that were fed into social media, and replicated or forwarded by interested users. Therefore, M-Eco was not the first instance to detect the public health event, because there were local actors who had already detected and reported about the event. But, M-Eco brought such reports quickly to a broader attention.

### SORMAS – Surveillance and outbreak management response system

SORMAS was a project among the Helmholtz Center for Infectious Research, Robert Koch-Institute, Bernhard-Nocht Institute and the Nigeria Field Epidemiology & Laboratory Training Program running from 2014 to 2015. It was started during the Ebola Virus Disease (EVD) outbreak in West Africa in August 2014 (Fähnrich et al. 2015; Adeoye et al. 2017). Despite the successful containment of the EVD outbreak in Nigeria at that time, the ongoing outbreaks in neighboring countries increased the potential for the introduction of new cases in Nigeria and other countries. Re-introduction of EVD in the community in Nigeria is of particular concern for further EVD spread given the population size and the high mobility of individuals living in Nigeria. No specific treatment or vaccine was available for EVD. Furthermore, EVD itself shows a variety of unspecific signs and symptoms at disease onset with a high risk of human-to-human transmission, which indicates the need for enhanced surveillance measures. Therefore and for the foreseeable future, the containment of the Ebola outbreak has to rely on a rapid and comprehensive identification of suspect cases, swift verification and assessment of contact persons. This signifies particular challenges in highly mobile populations living in areas with less reliable communication infrastructure and overwhelmed health care systems. SORMAS, the Surveillance and Outbreak Response Management System was developed to support:

- Transmission of (demographic) data to Ebola-infected at the national Nigerian Ebola Emergency Operation Center,
- Support the detection of symptomatic and demographic data on suspected cases and contacts,
- Management of the surveillance process of the contact persons of Ebola-infected.

It is a flexible outbreak management tool with real time data transfer. If also used in routine surveillance, it can bridge the gap between outbreak detection and response thus preparing for rapidly emerging infectious disease epidemics.

The technical concept of SORMAS system integrates existing technology and combines it into a mobile application, that allows to transmit notification reports, but also supports the whole management process of contact tracing. SORMAS was developed based on IMDB (in-memory database system) and cloud technology enabling task management on computers, smartphones and tablet devices. The user interface consists of specific front-ends for smartphones and tablet devices, which are independent from physical configurations. SORMAS allows real-time, bidirectional information exchange between field workers and the Emergency Operation Center, assures supervision of contact follow up, automated status reports, and GPS tracking.

Both categories of applications – apps for field workers incorporating mobile devices and apps for management personnel using desktop PCs – share the same platform and data storage, which enables interactive analysis of latest data without the need to export data into a dedicated On-Line Analytical Processing (OLAP) system.

Field workers use mobile devices, such as smartphones, to document acquired information directly in the cloud system. Available devices are registered in the cloud-based device management software SAP Afaria, which enables remote management of devices and user having access to the devices. This enables, for example, to keep device software automatically up-to-date and to track and wipe lost devices to ensure highest levels of data security. The local cellular phone network provider provides data transfer to the Internet. All data exchange is encrypted using latest web standards, e.g. HTTPS protocol. Applications are provided in a Software as a Service (SaaS) subscription model, i.e. all applications are configured, hosted, managed, and updated by the cloud service provider eliminating the need for any local IT management.

There is still development ongoing on SORMAS. An open source version SORMAS-open is available at https://github.com/hzi-braunschweig (last access: 23.07.2017). This version contains the “full workflow as the existing SORMAS in order to allow individual adaptations, to include a broader developer community and diminish apprehensions regarding unilateral economic dependencies” (http://www.sormas.org, last access: 18.07.2017).

## A model for assessing ethical risks of DDD

When running DDD projects or building DDD systems, it is relevant to consider and weight the different risks of the new technology against the benefits of its usage. For this reason, we developed an assessment model that aims at supporting in assessing the various ethical risks of a new DDD technology. Once aware of the different risks, it can be decided whether there are countermeasures available to reduce the risks or whether the risks are justifiable. Imagine a health status monitoring tool exploited by a health organization identifies a group of sick persons based on their social-media chatter. In which manner should the health organization react? Are they allowed to react? These and similar questions need to be answered before such applications go online. The model helps aims at revealing relevant aspects for DDD technologies and to raise such questions.

### The model

**Fig. 1:**
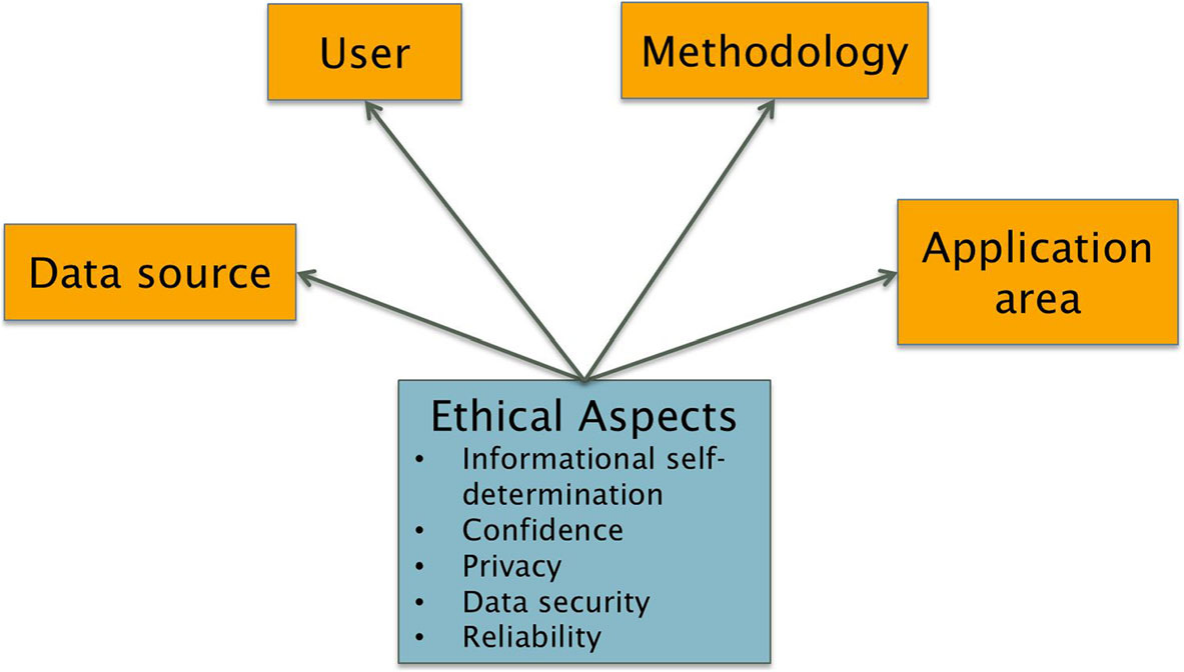
Assessment model comprises four aspects

To support the assessment of the ethical impact of digital epidemiology technologies, we suggest a novel model that comprises four aspects: user, application area, data source and methodology (see Fig. 1). For a concrete DDD application, it first needs to be clarified which users are involved, which application area is concerned and on which dimension it is operated. Questions include:

- Who is supposed to use the DDD system?
- Who is compelled to act on the new knowledge?
- What action is appropriate based on the information learned as a results of the analysis?
- Who is responsible when recognized information on a potential health threat is incorrect?

The user of a DDD system can be a health organization. However, when the systems provide results on publicly accessible websites, also by laymen can access. For example, MediSys (Linge et al. 2010) HealthMap (Brownstein et al. 2008) or other disease surveillance systems that collect and analyse data from the web are providing their results openly available through the web. Laymen normally do not have the background knowledge for correctly interpreting such data and a misinterpretation could cause panic in the population. Which unintended consequences might occur due to the availability of this data needs to be considered before making such data publicly available.

A second dimension is the application area. We can distinguish mainly four categories: monitoring, research (e.g. determining efficiency of vaccination campaigns), and communication. Within communication, applications concerning bi-directional communication among health officials (e.g. within SORMAS the data exchange among contact tracer and contact officers) and uni-directional communication from health officials to the public (e.g. informing the public on a crisis, vaccination campaigns) have to be considered separately. Regarding the application area, we have to distinguish primary and secondary usage of the results. This impacts the ethical issues concerning confidence, privacy, data and patient security or justice: While a research applications such as doing an epidemiological study on the spread of diseases using data collected from Internet sources can exploit anonymised data, other applications store personal data or require data that allows to contact individuals (e.g. SORMAS). Depending on the application area and the expected use or need of a DDD system, it is necessary to carefully weight harm and benefit for the individual or groups of persons Vayena et al. 2015. The assessment model shall help in reflecting the ethical issues of a technology and its application.

The data source has a large impact on the ethical risks. Data can originate from more official sources, but also from laymen, news agencies or others. Individuals report on disease symptoms. This can also include text messages from children or messages from persons recognizing disease activity in others (e.g. crowdsourcing). When registering for social media tools, a user agrees with the terms and conditions of the provider. However, an individual is not explicitly asked whether this data be exploited and analysed within a specific DDD system.

The type of data source impacts the reliability of the data. When the data is provided by individuals, it can be traced back to the individuals which is sometimes against the standard procedures of health organizations. Once individuals can be identified, there is a risk of social stigmatization. The user together with the source impacts on duties and responsibilities of who has to react and in which manner. Collected data need to be governed in ways that minimize the risk to harm individuals.

Methodology is the fourth dimension of the model. Among others, methods are required for managing biases, filtering systems for noisy data and selection of appropriate data streams. The dimension methodology includes the robustness of the scientific methodology, and the validation of algorithms (e.g. Did the algorithms have been validated before bringing into business?). When the methodology is error prone, there might be an increased risk of harm to individuals, businesses or communities if falsely detected as affected by infectious disease (e.g. tourist region, local minorities…).

### Application of the model to SORMAS and M-eco

The model supports in identifying and describing the ethical dimensions of a technology or use case and in identifying the ethical issues on the technology use from different perspectives. From the answers, ethical issues concerning confidence, privacy, data and patient security or justice may be judged and weighted. In this section, we demonstrate this application of the model on the example of M-Eco and SORMAS. Table 1 provides the basic aspects of the model related to the two systems introduced in section 3. We elaborate on them in the following.

**Table 1:** Model applied to SORMAS and M-Eco

	M-Eco	SORMAS
Data source / provider	- Everyone: official, news agencies, laymen	- Received through interviews
User	- Health organizations - Indirect: laymen	- Health officials
Methodology	- Frequency analysis - Filtering using machine learning - Internet	- Cloud services - OLAP data storage - Mobile phones
Application area	- Monitoring, disease surveillance - Early warning, risk reduction	- Communication and monitoring - Contact tracing - Outbreak management

The broad range of data sources exploited within M-Eco brings possibilities, but can cause also risks. First-hand information on disease development and symptoms becomes available, but on the other hand rumors can spread more easily. The reliability of the data need to be carefully reflected, since everyone can post through social media channels, one of the large sources of signal generation in M-Eco, and thus can spam the system and cause false alarms. The user must be aware of the potential risk of misinformation and the system could provide manual filtering options to exclude certain sources. Data from individuals posted in the Web can be traced backed, i.e. personal information becomes available. Before putting the system into daily business, guidelines need to be established that provide hints on how to react in those cases. Further, rules need to be established to avoid harm such as social discrimination or financial harm from the data provider.

The M-Eco platform itself was intended to be only accessible to registered users in health organizations. However, aggregated results are provided through the openly accessible MediSys system. Thus, depending on the signal, it could terrify people. Measurements need to be established on how to address these concerns. Regarding the methodology, filtering using machine learning influences the reliability, specificity and amount of data to be considered. When too much data is filtered, relevant signals might get lost, while having a broad filter risks overwhelming the user. Based on the specified application area, we can decide whether the data collection of the system is justifiable. According to the Helsinki declaration, only data relevant for the specified system can be stored.

In SORMAS, only official persons have access to the data. The data can be considered reliable, since it is collected in a face-to-face interview and through physical examination (measuring temperature, assessing symptoms). Implemented variable checks ensure that only reasonable data is stored. The data is only accessible to health officials. Through loss of the mobile phones, a subset of the data can become available (when no internet connection is available, the systems stores data locally until it can be uploaded). To avoid social stigmatization, people hide when contact tracer are coming. This risk could be determined early and campaigns for informing the population on the necessity of the data collection could help. The ethical concerns are mainly related to data privacy, since personal data is stored and refusing consent is actually inacceptable due to the high risk for large population groups. Measures could be to ensure that the data is stored safely and protected of being misused. Regarding data security, the laws of the country where the cloud server is placed need to be considered.

## Discussion of the application of the model

The two example show that the ethical assessment model helps to identify critical issues. Once the critical points are clear, countermeasures can be planned. We based the assessment model on existing guidelines and experiences gained from two DDD projects. There are other frameworks available. Kaas developed an ethics framework for public health (Kass 2001). It is not specifically designed for the new DDD technologies, but for public health interventions in general. The 6-step-framework considers 1) identification of the public health goals of a concrete intervention, 2) assessment of the effectiveness of the intervention in achieving the goals, 3) collecting the potential burdens of the intervention, and 4) specification of measures for minimizing these risks, 5) implementation of the intervention in a nondiscriminatory manner, 6) judgement whether the burdens and benefits can be balanced. These steps can be followed, once the relevant aspects are identified by our assessment model. The framework from Kass is missing concrete issues that are specific for DDD, e.g. data security, informational self-determination, data privacy.

Such issues are considered by the guidelines suggested by Mittelstadt. He identified ethical principles for designing the health-related Internet of Things and derived guidelines from these principles (Mittelstadt 2017). They concern individual and group privacy, trust and confidentiality, transparency of data protocols. As an important aspect, he claims that the user should allow how and which of his health data is used and analysed. In this context, there is a significant risk since data can be generated that “allow for unanticipated, invasive inferences about a user’s life” (Mittelstadt 2017).

The application of the suggested model does not result in direct answer whether a technologiy is ethically acceptable. Instead, it provides a mean to become aware of the ethical aspects of DDD already in the planning and development phase. It can be applied in an interdisciplinary meeting to collect different viewpoints on a DDD system and aims at triggering discussions and finding solutions for risks that might not be acceptable. Thus, it does not need necessarily be used when the DDD system has already been developed, but already in the phase of development. Only in that stage, we are able to still consider concerns and can develop in a way to better balance burdens and benefits. Even though it might be helpful to have concrete weights for judging ethical concerns, such weights would strongly depend on use case, public health goals of the DDD system.

## Conclusions

DDD is a new field in public health to gather health information that surely is not present in other more traditional and official sources of health information such as surveillance tools. Mobile devices and cloud technology provide new technological possibilities for disease surveillance and outbreak management. Counselling, confidentiality and privacy aspects are critical points to be considered in each case, following ethical and legal guidelines in the application of the medical profession. Social media or Internet data alone is rarely enough to confirm a public health problem. Linking with medical professionals to correlate and corroborate the findings from the data with actual medical data is important. However, health organisations or researchers must balance the rights of subjects with the social benefits of research. The model suggested in this paper helps in becoming aware of the ethical aspects. It can be applied in an interdisciplinary meeting to collect different viewpoints on a DDD system and aims at triggering discussions and finding solutions for risks that might not be acceptable. It is relevant to think on ethical aspects already in the development process (and possibly address them). In future work, we will verify the model and plan to create guidelines that allow to address these issues.

## Footnotes

1
http://www.oxforddictionaries.com/de/definition/englisch/ethics (last access 17.079.2017)

2
http://plato.stanford.edu/entries/publichealth-ethics/ (last access 10.07.2017)
